# 4-Chloro-2-[(*E*)-(4-nitro­phen­yl)diazenyl]phenol

**DOI:** 10.1107/S1600536809003675

**Published:** 2009-02-11

**Authors:** Leonid A. Aslanov, Ksenia A. Paseshnichenko, Alexandr V. Yatsenko

**Affiliations:** a Department of Chemistry, Moscow State University, 119992 Moscow, Russian Federation

## Abstract

The title compound, C_12_H_8_ClN_3_O_3_, in the crystalline state and in solution, exists in the azo form, as predicted by density functional theory (DFT) calculations. The mol­ecule is approximately planar [the dihedral angle between the rings is 1.83 (8)°], with the nitro group slightly twisted [13.4 (2)°] relative to the benzene ring. Translationally related mol­ecules form stacks along [010] with an inter­planar distance of 3.400 (2) Å. The hydroxy group forms an intramolecular hydrogen bond with the azo N atom.

## Related literature

For the crystal structure of a closely related mol­ecule, (1*Z*)-4-hydroxy­benzo-1,2-quinone-1-[(2-chloro-4-nitro­phen­yl)hydrazone, that crystallizes as a hydrazone tautomer, see: You *et al.* (2004 ▶). For reference structural data, see: Allen (2002 ▶). For details of the synthetic procedure, see: Fierz-David & Blangey (1949 ▶). For background on DFT calculations, see: Becke (1993 ▶); Klamt & Schüürmann (1993 ▶); Krishnan *et al.* (1980 ▶); Lee *et al.* (1988 ▶); Schmidt *et al.* (1993 ▶). For the concept of resonance-assisted hydrogen bonds, see: Gilli *et al.* (1989 ▶).
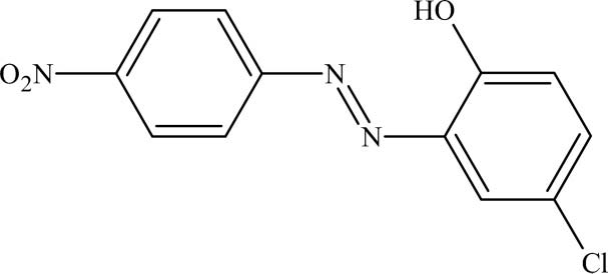


## Experimental

### 

#### Crystal data

C_12_H_8_ClN_3_O_3_
*M* *_r_* = 277.66
Monoclinic, 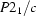

*a* = 19.008 (5) Å
*b* = 4.817 (2) Å
*c* = 12.862 (4) Å
β = 92.65 (2)°
*V* = 1176.4 (7) Å^3^
*Z* = 4
Mo *K*α radiation
μ = 0.33 mm^−1^
*T* = 291 (2) K
0.40 × 0.20 × 0.15 mm

#### Data collection

Enraf–Nonius CAD-4 diffractometer
Absorption correction: none
2567 measured reflections
2567 independent reflections
2110 reflections with *I* > 2σ(*I*)
3 standard reflections frequency: 90 min intensity decay: 4%

#### Refinement

*R*[*F* ^2^ > 2σ(*F* ^2^)] = 0.037
*wR*(*F* ^2^) = 0.098
*S* = 1.56
2567 reflections
173 parameters
H-atom parameters constrained
Δρ_max_ = 0.25 e Å^−3^
Δρ_min_ = −0.14 e Å^−3^

### 

Data collection: *CAD-4 Software* (Enraf–Nonius, 1989 ▶); cell refinement: *CAD-4 Software*; data reduction: *PROFIT* (Streltsov & Zavodnik, 1989 ▶) routine of *WinGX* (Farrugia, 1999 ▶); program(s) used to solve structure: *SHELXS97* (Sheldrick, 2008 ▶); program(s) used to refine structure: *SHELXL97* (Sheldrick, 2008 ▶); molecular graphics: *PLATON* (Spek, 2003 ▶); software used to prepare material for publication: *PLATON*.

## Supplementary Material

Crystal structure: contains datablocks global, I. DOI: 10.1107/S1600536809003675/gk2186sup1.cif

Structure factors: contains datablocks I. DOI: 10.1107/S1600536809003675/gk2186Isup2.hkl

Additional supplementary materials:  crystallographic information; 3D view; checkCIF report

## Figures and Tables

**Table 1:** Hydrogen-bond geometry (Å, °)

*D*—H⋯*A*	*D*—H	H⋯*A*	*D*⋯*A*	*D*—H⋯*A*
O1—H1⋯N2	0.82	1.88	2.5777 (17)	143

**Fig. 1.**
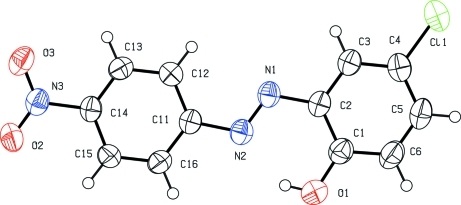
The molecular structure of the title compound with atomic labels and 50% probability displacement ellipsoids for non-H atoms.

**Fig. 2.**
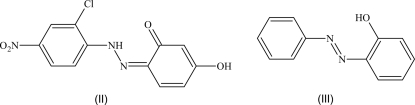
Chemical diagrams of (II) and (III).

## supplementary crystallographic information

### Comment

The present work was fulfilled in the course of study of the hydroxyazo-ketohydrazone tautomerism in phenylazophenols. The title compound, (I), exists in crystals as the azo form (Fig. 1). It is evidenced, firstly, by the fact that the H atom was found and refined in the vicinity of O atom, and secondly, by comparison of the molecular geometry with numerous structures of azo tautomers found in the Cambridge Structural Database (Allen, 2002). Since the UV–visible spectra of the crystalline title compound resemble its spectra in solutions, the azo tautomer has to predominate in solutions as well.

However, recently it has been reported that (1*Z*)-4-hydroxybenzo-1,2-quinone 1-[(2-chloro-4-nitrophenyl)hydrazone (II), the compound closely related to (I), exists in crystals as the hydrazone tautomer (You *et al.*, 2004).

The azo–hydrazone equilibrium is known to be shifted by the effect of donor and acceptor substituents and also by intra- and intermolecular hydrogen bonds. In order to evaluate the relative importance of these factors, we have performed the DFT calculations of azo and hydrazone tautomers of (I) and (II). Calculations were carried out using GAMESS (Schmidt *et al.*, 1993) with B3LYP exchange-correlation functional (Becke, 1993; Lee *et al.*, 1988) and 6-311G** basis set (Krishnan *et al.*, 1980). After geometry of an isolated molecule has been optimized, molecular structure was fixed, and the effect of nonspecific intermolecular interactions was accounted by COSMO method (Klamt & Schüürmann, 1993), taking the dielectric permeability equal to 10. The results indicate that for 2-phenyldiazenylphenol (III), the azo form is by 10.5 kJ/mol more stable than the hydrazone form. For compound (I), this difference decreases to 7.5 kJ/mol and for (II) - to 6.8 kJ/mol, but nonetheless the azo form is still preferable.

Thus, the difference between (I) and (II) most probably arises from specific intermolecular interactions. In (I), there is the only worthnoting intermolecular contact C15—H15···O2 (-*x*, -1 - *y*, -*z*) (H15···O2 2.56 Å, C15···O2 3.452 (2) Å, C15—H15···O2 161°), which cannot have any effect on the relative stability of tautomers. In (II), the keto group forms a strong hydrogen bond with the hydroxy group of a neighboring molecule (O···H 1.74 Å, O···O 2.581 (2) Å, O—H···O 173°). This interaction stabilizes the hydrazone tautomer, according to the conception of resonance-assisted hydrogen bonds (Gilli *et al.*, 1989). So, the shift of tautomeric equilibrium in (II) towards the hydrazone form should be most probably rationalized by formation of intermolecular hydrogen bonds.

### Experimental

The title compound was prepared by coupling of *p*-nitrophenyldiazonium chloride with *p*-chlorphenol. For details of the synthetic procedure, see Fierz-David & Blangey (1949). Single crystals were grown by slow evaporation of ethanol solution.

### Refinement

H atoms were located in a difference map and refined freely, but at final stage they were positioned geometrically and refined using a riding model with C—H = 0.93 Å, O—H = 0.82 Å and with *U*_iso_(H) = 1.2 times *U*_eq_(C), *U*_iso_(H) = 1.5 times *U*_eq_(O)

### Crystal data

**Table:**

C_12_H_8_ClN_3_O_3_	*F*(000) = 568
*M**_r_* = 277.66	*D*_x_ = 1.568 Mg m^−^^3^
Monoclinic, *P*2_1_/*c*	Mo *K*α radiation, λ = 0.71073 Å
Hall symbol: -P 2ybc	Cell parameters from 25 reflections
*a* = 19.008 (5) Å	θ = 16.8–18.8°
*b* = 4.817 (2) Å	µ = 0.33 mm^−^^1^
*c* = 12.862 (4) Å	*T* = 291 K
β = 92.65 (2)°	Prism, red
*V* = 1176.4 (7) Å^3^	0.40 × 0.20 × 0.15 mm
*Z* = 4	

### Data collection

**Table:**

Enraf–Nonius CAD-4 diffractometer	*R*_int_ = 0.0
Radiation source: fine-focus sealed tube	θ_max_ = 27.0°, θ_min_ = 1.1°
graphite	*h* = −24→24
nonprofiled ω scans	*k* = 0→6
2567 measured reflections	*l* = 0→16
2567 independent reflections	3 standard reflections every 90 min
2110 reflections with *I* > 2σ(*I*)	intensity decay: 4%

### Refinement

**Table:**

Refinement on *F*^2^	Primary atom site location: structure-invariant direct methods
Least-squares matrix: full	Secondary atom site location: difference Fourier map
*R*[*F*^2^ > 2σ(*F*^2^)] = 0.037	Hydrogen site location: inferred from neighbouring sites
*wR*(*F*^2^) = 0.098	H-atom parameters constrained
*S* = 1.56	*w* = 1/[σ^2^(*F*_o_^2^) + (0.04*P*)^2^] where *P* = (*F*_o_^2^ + 2*F*_c_^2^)/3
2567 reflections	(Δ/σ)_max_ = 0.001
173 parameters	Δρ_max_ = 0.25 e Å^−^^3^
0 restraints	Δρ_min_ = −0.14 e Å^−^^3^

### Special details

**Table:**

Geometry. All e.s.d.'s (except the e.s.d. in the dihedral angle between two l.s. planes) are estimated using the full covariance matrix. The cell e.s.d.'s are taken into account individually in the estimation of e.s.d.'s in distances, angles and torsion angles; correlations between e.s.d.'s in cell parameters are only used when they are defined by crystal symmetry. An approximate (isotropic) treatment of cell e.s.d.'s is used for estimating e.s.d.'s involving l.s. planes.
Refinement. Refinement of *F*^2^ against ALL reflections. The weighted *R*-factor *wR* and goodness of fit *S* are based on *F*^2^, conventional *R*-factors *R* are based on *F*, with *F* set to zero for negative *F*^2^. The threshold expression of *F*^2^ > σ(*F*^2^) is used only for calculating *R*-factors(gt) *etc*. and is not relevant to the choice of reflections for refinement. *R*-factors based on *F*^2^ are statistically about twice as large as those based on *F*, and *R*- factors based on ALL data will be even larger.

### Fractional atomic coordinates and isotropic or equivalent isotropic displacement parameters (Å^2^)

**Table:**

	*x*	*y*	*z*	*U*_iso_*/*U*_eq_	
Cl1	0.463076 (19)	1.28010 (9)	0.16237 (3)	0.06275 (16)	
O1	0.27449 (6)	0.7166 (3)	−0.14202 (8)	0.0703 (4)	
H1	0.2491	0.6045	−0.1138	0.105*	
O2	−0.00077 (6)	−0.4250 (3)	0.11633 (9)	0.0665 (3)	
O3	0.05796 (6)	−0.4261 (2)	0.26393 (8)	0.0592 (3)	
N1	0.27334 (5)	0.5696 (2)	0.07974 (9)	0.0436 (3)	
N2	0.23136 (6)	0.4462 (2)	0.01583 (9)	0.0450 (3)	
N3	0.04760 (6)	−0.3453 (2)	0.17452 (10)	0.0468 (3)	
C1	0.31710 (7)	0.8384 (3)	−0.07021 (11)	0.0496 (4)	
C2	0.31686 (6)	0.7693 (3)	0.03656 (10)	0.0420 (3)	
C3	0.36225 (7)	0.9076 (3)	0.10740 (11)	0.0444 (3)	
H3	0.3620	0.8642	0.1778	0.053*	
C4	0.40705 (7)	1.1065 (3)	0.07365 (12)	0.0475 (3)	
C5	0.40778 (8)	1.1751 (3)	−0.03104 (12)	0.0556 (4)	
H5	0.4387	1.3097	−0.0534	0.067*	
C6	0.36281 (8)	1.0440 (4)	−0.10150 (12)	0.0589 (4)	
H6	0.3629	1.0935	−0.1714	0.071*	
C11	0.18678 (6)	0.2481 (3)	0.06134 (10)	0.0402 (3)	
C12	0.18713 (7)	0.1938 (3)	0.16816 (11)	0.0472 (3)	
H12	0.2177	0.2884	0.2143	0.057*	
C13	0.14144 (7)	−0.0020 (3)	0.20368 (10)	0.0473 (3)	
H13	0.1412	−0.0428	0.2743	0.057*	
C14	0.09613 (6)	−0.1372 (3)	0.13447 (10)	0.0401 (3)	
C15	0.09456 (7)	−0.0863 (3)	0.02927 (10)	0.0456 (3)	
H15	0.0634	−0.1801	−0.0161	0.055*	
C16	0.14084 (7)	0.1087 (3)	−0.00691 (11)	0.0469 (3)	
H16	0.1411	0.1465	−0.0778	0.056*	

### Atomic displacement parameters (Å^2^)

**Table:**

	*U*^11^	*U*^22^	*U*^33^	*U*^12^	*U*^13^	*U*^23^
Cl1	0.0544 (2)	0.0552 (3)	0.0784 (3)	−0.00993 (18)	0.00095 (19)	−0.0052 (2)
O1	0.0844 (8)	0.0812 (9)	0.0452 (6)	−0.0262 (7)	0.0024 (5)	0.0036 (6)
O2	0.0716 (7)	0.0648 (8)	0.0636 (7)	−0.0297 (6)	0.0068 (6)	−0.0067 (6)
O3	0.0716 (7)	0.0500 (6)	0.0570 (7)	−0.0031 (5)	0.0129 (5)	0.0118 (5)
N1	0.0437 (6)	0.0373 (6)	0.0502 (6)	0.0005 (5)	0.0071 (5)	0.0018 (5)
N2	0.0460 (6)	0.0406 (6)	0.0486 (6)	−0.0002 (5)	0.0054 (5)	0.0021 (5)
N3	0.0548 (7)	0.0348 (6)	0.0518 (7)	0.0001 (5)	0.0128 (5)	−0.0031 (5)
C1	0.0523 (8)	0.0494 (8)	0.0477 (8)	0.0001 (7)	0.0095 (6)	0.0004 (7)
C2	0.0418 (6)	0.0367 (7)	0.0482 (8)	0.0030 (6)	0.0092 (6)	0.0027 (6)
C3	0.0452 (7)	0.0382 (7)	0.0502 (7)	0.0040 (6)	0.0072 (6)	0.0035 (6)
C4	0.0432 (7)	0.0373 (7)	0.0626 (9)	0.0023 (6)	0.0067 (6)	−0.0042 (7)
C5	0.0551 (8)	0.0473 (9)	0.0663 (10)	−0.0056 (7)	0.0214 (7)	0.0042 (8)
C6	0.0663 (9)	0.0596 (10)	0.0522 (9)	−0.0060 (8)	0.0176 (7)	0.0075 (8)
C11	0.0402 (6)	0.0351 (7)	0.0456 (7)	0.0033 (5)	0.0055 (5)	0.0018 (6)
C12	0.0478 (7)	0.0485 (8)	0.0451 (8)	−0.0054 (6)	0.0000 (6)	−0.0017 (6)
C13	0.0537 (8)	0.0501 (8)	0.0384 (7)	−0.0021 (7)	0.0041 (6)	0.0024 (6)
C14	0.0419 (6)	0.0296 (6)	0.0492 (7)	0.0025 (5)	0.0081 (5)	−0.0005 (6)
C15	0.0505 (7)	0.0426 (8)	0.0432 (7)	−0.0058 (6)	−0.0015 (6)	−0.0025 (6)
C16	0.0540 (8)	0.0472 (8)	0.0395 (7)	−0.0023 (6)	0.0030 (6)	0.0053 (6)

### Geometric parameters (Å, °)

**Table:**

Cl1—C4	1.7387 (16)	C4—C5	1.387 (2)
O1—C1	1.3358 (17)	C5—C6	1.371 (2)
O1—H1	0.8200	C5—H5	0.9300
O2—N3	1.2203 (15)	C6—H6	0.9300
O3—N3	1.2214 (15)	C11—C16	1.3831 (18)
N1—N2	1.2670 (16)	C11—C12	1.3983 (19)
N1—C2	1.4001 (17)	C12—C13	1.3743 (19)
N2—C11	1.4203 (17)	C12—H12	0.9300
N3—C14	1.4712 (17)	C13—C14	1.3741 (19)
C1—C6	1.389 (2)	C13—H13	0.9300
C1—C2	1.413 (2)	C14—C15	1.3741 (19)
C2—C3	1.3949 (19)	C15—C16	1.3823 (19)
C3—C4	1.3660 (19)	C15—H15	0.9300
C3—H3	0.9300	C16—H16	0.9300
			
C1—O1—H1	109.5	C5—C6—C1	121.06 (14)
N2—N1—C2	115.52 (11)	C5—C6—H6	119.5
N1—N2—C11	114.69 (11)	C1—C6—H6	119.5
O2—N3—O3	124.14 (12)	C16—C11—C12	120.47 (12)
O2—N3—C14	117.79 (12)	C16—C11—N2	115.84 (12)
O3—N3—C14	118.06 (12)	C12—C11—N2	123.69 (12)
O1—C1—C6	118.70 (13)	C13—C12—C11	118.72 (13)
O1—C1—C2	122.63 (13)	C13—C12—H12	120.6
C6—C1—C2	118.67 (14)	C11—C12—H12	120.6
C3—C2—N1	115.33 (12)	C14—C13—C12	119.79 (12)
C3—C2—C1	119.50 (13)	C14—C13—H13	120.1
N1—C2—C1	125.17 (13)	C12—C13—H13	120.1
C4—C3—C2	120.21 (13)	C13—C14—C15	122.53 (12)
C4—C3—H3	119.9	C13—C14—N3	118.73 (12)
C2—C3—H3	119.9	C15—C14—N3	118.75 (12)
C3—C4—C5	120.66 (14)	C14—C15—C16	117.88 (12)
C3—C4—Cl1	120.05 (12)	C14—C15—H15	121.1
C5—C4—Cl1	119.29 (11)	C16—C15—H15	121.1
C6—C5—C4	119.88 (14)	C15—C16—C11	120.61 (12)
C6—C5—H5	120.1	C15—C16—H16	119.7
C4—C5—H5	120.1	C11—C16—H16	119.7
			
C2—N1—N2—C11	−178.90 (10)	N1—N2—C11—C16	−179.47 (11)
N2—N1—C2—C3	179.08 (11)	N1—N2—C11—C12	1.27 (18)
N2—N1—C2—C1	−0.30 (19)	C16—C11—C12—C13	0.5 (2)
O1—C1—C2—C3	−179.22 (13)	N2—C11—C12—C13	179.77 (12)
C6—C1—C2—C3	−0.1 (2)	C11—C12—C13—C14	−0.7 (2)
O1—C1—C2—N1	0.1 (2)	C12—C13—C14—C15	0.4 (2)
C6—C1—C2—N1	179.24 (13)	C12—C13—C14—N3	−179.82 (12)
N1—C2—C3—C4	179.97 (11)	O2—N3—C14—C13	166.51 (12)
C1—C2—C3—C4	−0.6 (2)	O3—N3—C14—C13	−13.07 (17)
C2—C3—C4—C5	0.4 (2)	O2—N3—C14—C15	−13.72 (18)
C2—C3—C4—Cl1	179.53 (10)	O3—N3—C14—C15	166.71 (12)
C3—C4—C5—C6	0.5 (2)	C13—C14—C15—C16	0.1 (2)
Cl1—C4—C5—C6	−178.60 (12)	N3—C14—C15—C16	−179.63 (11)
C4—C5—C6—C1	−1.3 (2)	C14—C15—C16—C11	−0.3 (2)
O1—C1—C6—C5	−179.81 (14)	C12—C11—C16—C15	0.0 (2)
C2—C1—C6—C5	1.1 (2)	N2—C11—C16—C15	−179.28 (12)

### Hydrogen-bond geometry (Å, °)

**Table:**

*D*—H···*A*	*D*—H	H···*A*	*D*···*A*	*D*—H···*A*
O1—H1···N2	0.82	1.88	2.5777 (17)	143
